# Using a Mobile App-Based International Classification of Functioning, Disability, and Health Set to Assess the Functioning of Spinal Cord Injury Patients: Rasch Analysis

**DOI:** 10.2196/20723

**Published:** 2020-11-11

**Authors:** Mengmeng Jia, Jie Tang, Sumei Xie, Xiaokuo He, Yingmin Wang, Ting Liu, Tiebin Yan, Kun Li

**Affiliations:** 1 School of Nursing Sun Yat-sen University Guangzhou China; 2 Department of Spinal Cord Injury Rehabilitation Sichuan Provincial Rehabilitation Hospital Chengdu China; 3 Department of Spinal Cord Injury Rehabilitation Guangdong Provincial Work Injury Rehabilitation Hospital Guangzhou China; 4 Department of Rehabilitation Medicine The Fifth Hospital of Xiamen Xiamen China; 5 Department of Rehabilitation Medicine Sun Yat-sen Memorial Hospital Guangzhou China

**Keywords:** International Classification of Functioning, Disability and Health, spinal cord injuries, mobile health app, Rasch analysis

## Abstract

**Background:**

The International Classification of Functioning, Disability, and Health (ICF) is a unified system of functioning terminology that has been used to develop electronic health records and assessment instruments. Its application has been limited, however, by its complex terminology, numerous categories, uncertain operationalization, and the training required to use it well. Together is a mobile health app designed to extend medical support to the families of spinal cord injury (SCI) patients in China. The app’s core framework is a set of only 31 ICF categories. The app also provides rating guidelines and automatically transforms routine assessment results to the terms of the ICF qualifiers.

**Objective:**

The goal of the research is to examine the suitability of the ICF set used in the app Together for use as an instrument for assessing the functioning of SCI patients.

**Methods:**

A cross-sectional study was conducted including 112 SCI patients recruited before discharge from four rehabilitation centers in China between May 2018 and October 2019. Nurses used the app to assess patient functioning in face-to-face interviews. The resulting data were then subjected to Rasch analysis.

**Results:**

After deleting two categories (family relationships and socializing) and one personal factor (knowledge about spinal cord injury) that did not fit the Rasch model, the body functions and body structures, activities and participation, and contextual factors components of the ICF exhibited adequate fit to the Rasch model. All three demonstrated acceptable person separation indices. The 28 categories retained in the set were free of differential item functioning by gender, age, education level, or etiology.

**Conclusions:**

Together overcomes some of the obstacles to practical application of the ICF. The app is a reliable assessment tool for assessing functioning after spinal cord injury.

## Introduction

The International Classification of Functioning, Disability, and Health (ICF) is a unified system of terminology for multidisciplinary use issued in 2001 by the World Health Organization (WHO). It provides a consensus framework for defining functioning and disability and their interrelationships with health conditions and contextual factors [1]. As a standard language, the ICF is designed to be easily understood and used among multidisciplinary teams [2]. The comprehensive perspective on functioning and interdisciplinary focus have motivated the development of data collection tools, electronic health records, and assessment instruments [3-5], and it is now sometimes viewed as a third health indicator for monitoring a health system’s performance after mortality and morbidity [6]. Many studies have provided evidence of the ICF’s value in reflecting patient levels of functioning, helping decision making, enhancing collaboration, and planning treatment.

However, challenges have limited practical application of the ICF, including its relatively complex terminology and category numbering. Each ICF category has its own distinct definition, which doesn’t always accord with the prevailing medical terminology. Professionals need to be trained before using the system [5]. Although many ICF core sets with fewer categories have been specifically developed for certain conditions, some studies still report that the application of the ICF is time-consuming [7]. Reducing the number of categories by selecting only the most relevant remains challenging. Additionally, rating using the ICF qualifiers is not easy. There are 5 grades: 0 = no problem, 1 = mild, 2 = moderate, 3 = severe, and 4 = complete problem. But there can be large differences among user assessments because of a lack of clear assessment guidelines, giving the approach poor interrater reliability [8-11]. Some researchers have suggested establishing assessment guidelines, but these would necessarily be complex and require additional user training, further hindering the system’s acceptance [12,13].

A mobile health app is a health-related software program installed on smart mobile devices that provides health information and tracks a user’s health behavior. It might also allow remote consultation [14]. With the popularity of personal mobile devices such as cellphones and tablets, mobile apps have been used in many fields in health care [15,16]. Studies have shown that app-based transitional care enhances patients’ self-perceptions of efficacy [17], helps prevent complications [18,19], improves quality of life [18], and reduces readmissions to hospital [18].

The study team previously developed an ICF-based app called Together for the transitional care of spinal cord injury (SCI) patients in China [20]. SCI is a serious and life-changing disease that can cause paraplegia or quadriplegia. Most SCI patients in China live at home after their acute treatment and rehabilitation [21]. They almost always need professional medical support for further rehabilitation and preventing complications [22,23]. However, community medical resources in China are at present limited and cannot meet SCI patients’ complex long-term health needs [24]. Together was designed to bring professional health care support from medical institutions to SCI patients living with their families in China. The language of the app is Chinese, and the copyright is held by China’s Sun Yat-sen University [25].

Together’s core framework is a set of ICF categories that reflect the levels of functioning typical of SCI patients and help organize online assessment, standardize health guidance, and coordinate interdisciplinary collaboration. The app uses fewer and more specific categories than the normal ICF core set, focusing on the transitional care of SCI patients. Preliminary studies identified 31 ICF categories as the most useful outcome indicators in the transitional care of SCI patients, covering the major physiological, psychological, and social participation problems of SCI patients at home [20,26]. The app provides consistent assessment prompts for the raters. Guidelines are provided for rating each ICF category, which helps to guarantee the consistency of the assessments, minimize the training required, and ease the load on the clinical staff doing the assessments.

This study was part of a research program designed to document the effects of an app-based transitional care model for SCI patients at home. Rasch analysis was applied to examine the suitability of the app’s set of categories. The overall aim was to determine to what extent Together can solve problems related to using the ICF in clinical practice.

The assumption of the Rasch model is that a person with greater ability is more likely than a person with less ability to pass in relation to an item, and that an easy item is more likely to be passed than a difficult one [27]. A Rasch analysis is used to examine whether an instrument makes those distinctions satisfactorily. A person’s performance on an item should be related only to the person’s ability and the difficulty of the item, regardless of gender, age, education, etc. [27]. If an instrument fits the Rasch model well, it can be used to reflect the performance of people with different abilities. This study was designed to test Together’s performance in that regard. A good result could present a new approach in the use of the ICF.

## Methods

### Study Design

A cross-sectional design was employed involving four research centers in Guangzhou, Chengdu, and Shiyan in China. The study was approved by Sun Yat-sen University’s ethics committee (file 2017ZSLYEC-0620).

### Participants

The participants were recruited between May 2018 and October 2019 prior to discharge from the four research centers. The inclusion criteria were age 18 years or older, SCI according to the International Standards for Neurological Classification of Spinal Cord Injury [28] and imaging examination, less than 2 years since injury, conscious and able to communicate, and in possession of an internet-connected mobile device and familiarity with using it. The exclusion criteria were severe heart, brain, lung, liver, or kidney disease; acute-stage spinal cord injury or in the critical period; or spinal cord lesions with a degenerative, genetic, or congenital cause.

### App

The Together app was designed to help medical staff provide remote follow-up for home-dwelling SCI patients. Hospital-based nurses, physicians, and therapists responsible for the transitional care for SCI patients at home are the target users. The core functions of the app comprise online assessment, providing standard health guidance, interdisciplinary referral within the team, interaction among health staff and patients, and management of online follow-up. With the help of the app, health care personnel can assess patient performance in terms of ICF categories and remotely provide health education to patients according to the assessment results. The app can be used to refer patients to different professionals on the health care team. Weekly reminders make managing follow-up by medical staff easier.

The app’s development has been reported previously [20,26]. From 51 ICF categories identified as outcome indicators useful in the transitional care of SCI patients by experts in the field via a 3-round Delphi survey, 31 were selected by a panel of 5 experts to form the app’s framework based on the feasibility of use in clinical practice. them. These categories best reflected the dysfunctions and complications most common and most in need of monitoring for SCI patients at home. Categories reflecting actual performance of SCI patients in daily life were preferred.

The 31 ICF categories address physiological functioning, psychological functioning, complications, daily living activities, social participation, adaptation to environmental factors, and personal factors. For each category, guidelines were established for converting routine clinical assessment results to the ICF qualifiers, and a standardized guidance program was formulated by the expert panel based on the knowledge-attitude-practice theory. Together is an Android app developed using the Java language. Most of the app’s functions—online assessment, providing standard health guidance, interdisciplinary referral—apply to all of the categories.

**Figure 1:**
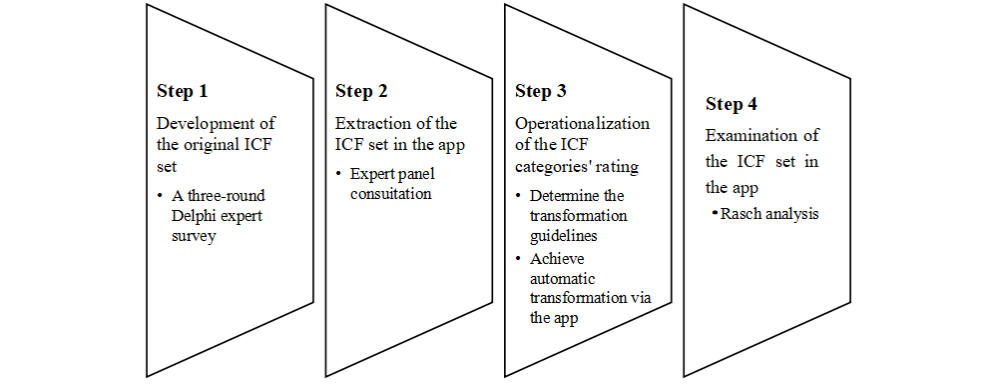
Development process of the Together app.

The app’s utility depends heavily on to what extent its assessment results reflect functioning differences among patients with different capabilities. This study used Rasch analysis to test the app’s suitability as an assessment instrument. Figure 1 shows the development and examination process.

### Assessment Guidelines

Medical staff communicate with the patients face to face or by telephone, assess them on each ICF category, and record the results in the app, which offers standard verbal prompts to the clinician to unify different clinicians’ assessments with respect to each ICF category. For example, the verbal prompt of the sensation of pain (b280) category was “If 0 is not painful and 10 is the most painful, how serious is your pain?” Four transformation guidelines were developed to transform the initial clinical assessment results: 0 = no problem, 1 = mild, 2 = moderate, 3 = severe, or 4 = complete problem (Table 1) [29]. The clinician is responsible for the initial assessments and the app automatically transforms their initial assessment results to the ICF qualifiers according to the preset guidelines.

Guideline 1 transforms patient information in the form of percentages to the ICF qualifiers. Using muscle power functions (b730) as an example, the 0 qualifier would indicate that all of the key muscles below the injured neurological level had power grade >3, and the 4 qualifier would indicate that 95% to 100% of key muscles had power grade <3. Guideline 2 transforms the wording of patient reports to the qualifiers. Using mobility of joint functions (b710) as an example, no limitation of joint mobility, slight limitation, moderate limitation, severe limitation, and total immobility would be rated as 0, 1, 2, 3, and 4, respectively. Guideline 3 transforms the frequency with which a problem was observed to the qualifiers. Using increased blood pressure (b4200) as an example, stable blood pressure over the past month would be rated as 0, whereas high blood pressure almost every day would receive a 4. Guideline 4 transforms assessment results generated using routine clinical instruments or standards, such as the 0-10 numerical rating scale (NRS) for pain, to the qualifiers. NRS scores of 0, 1-2, 3-4, 5-9, and 10 would be rated as 0, 1, 2, 3, and 4, respectively (Multimedia Appendix 1).

**Table 1:** Guidelines for transforming routine assessment results to the International Classification of Functioning, Disability, and Health qualifiers.

ICF^a^ qualifier	Guideline 1^b^	Guideline 2^c^	Guideline 3^d^	Guideline 4^e^
0 (no problem)	0%-4%	No, none, absent, negligible...	The person has no such problem.	NRS^f^ for pain, MAS^g^, NPIAP^h^ stage, FIM^i^, WHOQoL-BREF^j^, SF-36^k^
1 (mild problem)	5%-24%	Mild, slight, low...	The problem rarely happened in the last month (<25% of the time).	NRS^f^ for pain, MAS^g^, NPIAP^h^ stage, FIM^i^, WHOQoL-BREF^j^, SF-36^k^
2 (moderate problem)	25%-49%	Moderate, medium, fair...	The problem happened occasionally in the last month (<50% of the time).	NRS^f^ for pain, MAS^g^, NPIAP^h^ stage, FIM^i^, WHOQoL-BREF^j^, SF-36^k^
3 (severe problem)	50%-95%	Severe, high, extreme...	The problem happened frequently in the last month (>50% of the time).	NRS^f^ for pain, MAS^g^, NPIAP^h^ stage, FIM^i^, WHOQoL-BREF^j^, SF-36^k^
4 (complete problem)	96%-100%	Complete, total...	The problem happened almost every day in the last month (>95% of the time).	NRS^f^ for pain, MAS^g^, NPIAP^h^ stage, FIM^i^, WHOQoL-BREF^j^, SF-36^k^

^a^ICF: International Classification of Functioning, Disability, and Health.

^b^Guideline 1: transforms patient information in the form of percentages to the ICF qualifiers.

^c^Guideline 2: transforms wording from patient reports to the ICF qualifiers.

^d^Guideline 3: transforms the frequency with which a problem was observed during the previous month to the ICF qualifiers.

^e^Guideline 4: transforms the scores of a routine clinical instrument or standards to the ICF qualifiers.

^f^NRS: numeric rating scale.

^g^MAS: Modified Ashworth Scale.

^h^NPIAP: National Pressure Injury Advisory Panel.

^i^FIM: Functional Independence Measure.

^j^WHOQoL-BREF: World Health Organization Quality of Life Assessment–Abbreviated.

^k^SF-36: 36-Item Short Form Health Survey.

### Instruments

#### Demographic and Disease Questionnaire

The questionnaire consisted of two parts, including demographic and disease-related data such as name, gender, age, education level, diagnosis, etiology, American Spinal Injury Association Impairment Scale grade, SCI level, and duration of the disability. The information was collected by nurses in face-to-face interviews with the patients and by reviewing their medical records.

#### App-Based International Classification of Functioning, Disability, and Health Set

The 31 categories came from the three components body functions and body structures (15 categories), activities and participation (10 categories), and contextual factors (6 categories). Although the ICF does not classify personal factors in the contextual factors component, four personal factor items related to the psychology of SCI patients were included based on preliminary testing (acceptance of life in a wheelchair/in bed, knowledge about spinal cord injury, coping with everyday life, and adjustment to new body image) [20,26]. The app was then used to rate the SCI patients’ functioning on a 1-4 scale. In addition, an option 9 (not applicable) was also used. For the activities and participation component, each patient’s performance on tasks in actual life situations (not in a standard environment) was assessed. For the contextual factors component, barriers (but not facilitators) encountered in each patient’s life were assessed. In previous research, the eating (d550) and drinking (d560) categories were found to be strongly interrelated and difficult to assess separately [13]; therefore, those categories were combined into a testlet to be assessed.

### Data Collection

All nurses who participated in the study first received half a day of training involving a lecture and workshop, including an introduction to the study and how to use the app. Eligible patients were invited to participate in the study before discharge. After signing the informed consent form, demographic and disease-related data were collected by the trained nurses in face-to-face interviews and by reviewing the patients’ medical records. The nurses then assessed the patients’ performance with respect to each ICF category using the app. They did this face to face referring to the standard verbal prompts for each category in the app. The app system allows submission only after all categories have been evaluated; otherwise, the system indicates to the user that the evaluation is incomplete.

### Analysis

#### Data Processing

SPSS Statistics software version 21.0 (IBM Corporation) was used to analyze the demographic and disease data. RUMM2030 software (RUMM Laboratory Pty) was used to perform the Rasch analysis. For each component of the ICF set, the overall fit to a Rasch model was examined. If the overall fit was not good, poorly fitting categories were identified and deleted. Another round of Rasch analysis was then run until adequate overall fit was attained. The following properties of the ICF set were examined.

#### Overall Fit to the Rasch Model

A nonsignificant value in a χ^2^ test for item-trait interaction, a mean within ±2.5 (SD <1.5) for the fit residuals of the items and persons indicate good overall fit to the Rasch model [30]. Fit residuals represent the extent to which the observations do not fit a Rasch model. The significance level was adjusted using the Bonferroni correction [31].

#### Single-Item Fit to the Rasch Model

The good fit of a single category was represented by a nonsignificant χ^2^ test and a mean of the fit residual values within ±2.5 [32].

#### Person Separation Index

An acceptable person separation index indicates good internal consistency for the instrument and reflects the ability of the instrument to discriminate between people with different abilities. It has a range of 0 to 1, with higher values indicating a better ability (>0.7 indicates good) [33].

#### Differential Item Functioning

For an ideal Rasch model, no factors should influence a person’s performance regarding an item except the Rasch factors [30]. The differential item functioning shows the influences of the other factors. In this study, a nonsignificant analysis of variance result was taken as indicating no differential item functioning for a specific category based on gender, age, education level, or etiology.

## Results

### Patient Characteristics

The demographic and disease characteristics of all 112 spinal cord injury patients are shown in Table 2. Their ages ranged from 18 to 65 years (mean 41.7 [SD 12.3]); 82.1% (92/112) of the patients were younger than 60 years, with patients in the 40- to 49-year age group the most numerous; 83.0% (93/112) of the patients were male. A total of 60.8% (68/112) claimed to have had a middle school education and 25.9% (29/112) only primary education or less. The duration of their disability ranged from 1 to 22 months (mean 7.1 [SD 4.2]); 88.4% (99/112) had been injured for less than a year. Most of the injuries (100/112, 89.3%) were caused by trauma. Most of the patients were injured at the thoracic and cervical levels, accounting for 50.0% (56/112) and 27.7% (31/112), respectively. About half (57/112, 50.9%) of the patients had complete injury.

**Table 2:** Characteristics of the study sample (n=112).

Characteristic		Value, n (%)
**Gender**		
	Male	93 (83.0)
	Female	19 (17.0)
**Age in years**		
	18-29	27 (24.1)
	30-39	19 (17.0)
	40-49	36 (32.1)
	50-59	20 (17.9)
	60-65	20 (17.9)
**Education**		
	Primary school and below	29 (25.9)
	Junior high school	47 (42.0)
	Senior high school	21 (18.8)
	College and above	15 (13.4)
**Etiology**		
	Trauma	100 (89.3)
	Nontrauma	12 (10.7)
**Duration of disease in months**		
	1-6	59 (52.7)
	7-12	40 (35.7)
	13-18	12 (10.7)
	19-22	1 (0.9)
**American Spinal Injury Association** **Impairment scale**		
	Complete injury	57 (50.9)
	Incomplete injury	55 (49.1)
**Spinal cord injury** **level**		
	Cervical	31 (27.7)
	Thoracic	56 (50.0)
	Lumbar sacral	25 (22.3)

### Rasch Analysis Results

The 31 ICF categories belonged to body functions and body structures (15), activities and participation (10), and contextual factors (6). To attain adequate fit to the Rasch model for each component, categories that did not fit were deleted as multiple rounds of Rasch analysis were conducted. Table 3 shows the process and results of the Rasch analysis for each component.

**Table 3:** Summary of results of the Rasch analyses (n=112).

Analysis and action			Item fit residual, mean (SD)	Person fit residual, mean (SD)	Overall model fit^a^		Person separation index
					χ^2^	*P* value	
**Body functions and body structures**							
	1	Original categories	–0.41 (0.86)	–0.32 (0.62)	41.5	.08	0.50
**Activities and participation**							
	2	Original categories	–0.48 (2.27)	–0.38 (0.80)	75.7	<.001^b^	0.89
	3	Deleted family relationships (d760)	–0.18 (2.37)	–0.30 (0.79)	43.3	<.001^b^	0.89
	6	Deleted family relationships (d760) and socializing (d9205)	–0.06 (1.59)	–0.23 (0.73)	24.7	.08	0.89
**Environmental factors and personal factors**							
	7	Original categories	0.25 (1.63)	–0.45 (1.30)	32.8	.001^b^	0.65
	8	Deleted knowledge about spinal cord injury	–0.33 (1.32)	–0.43 (1.22)	13.6	.19	0.68

^a^Overall model fit was tested using a χ^2^ test with a Bonferroni-adjusted *P* value. The values were all *P*<.01.

^b^Significant according to the Bonferroni-adjusted *P* value.

In the first-round Rasch analysis, the body functions and body structures component consisting of 15 categories exhibited a nonsignificant χ^2^ test result for the item-trait interaction (χ^2^_30_=41.5, *P*=.08, Bonferroni-adjusted *P*=.05/15=.003). Additionally, the means for the item and person fit residuals were within ±2.5 (SD <1.5). These results suggested a good fit to the Rasch model. All of the 15 categories exhibited nonsignificant χ^2^ test results and the means of their fit residual values were also within acceptable limits (Table 4). The person separation index of this component was 0.5. There was no differential item functioning for any of the categories by gender, age, education level, or etiology.

Regarding the initial activities and participation component with 10 categories, in the first-round Rasch analysis, the χ^2^ test for the item-trait interaction yielded a significant result (χ^2^_20_=75.7, *P*<.001, Bonferroni-adjusted *P*=.05/10=.005). The single-item fit analysis found that four categories, changing basic body position (d410), washing oneself (d510), family relationships (d760), and socializing (d9205), did not fit the Rasch model. The family relationships (d760) and socializing (d9205) categories both exhibited poor fit results in a previous study [13]. Considering the mean fit residual values 3.253 for family relationships (d760) and 2.605 for socializing (d9205) and the category meanings, d760 was deleted first. However, the χ^2^ test result for the item-trait interaction in the second-round Rasch analysis (after deleting d760) remained significant (χ^2^_18_=43.3, *P*<.001, Bonferroni-adjusted *P*=.05/9=.0056). A third round of Rasch analysis was performed after deleting both family relationships (d760) and socializing (d9205). Although the standard deviation of the overall item fit residuals (SD 1.59) was a little larger than the upper limit, the χ^2^ test result for the item-trait interaction was no longer significant (χ^2^_16_=24.7, *P*=.08, Bonferroni-adjusted *P*=.05/8=.0063), suggesting good fit to the Rasch model. The single-item fit tests for the remaining 8 categories also yielded good model fit results, with nonsignificant χ^2^ test results (Table 4). The person separation index of the component was excellent (0.89) and no differential item functioning was detected for any of the categories by gender, age, education level, or etiology.

For the contextual factors component, the first-round Rasch analysis starting with 6 categories indicated poor model fit according to the χ^2^ test result for the item-trait interaction (χ^2^_12_=32.8, *P*<.001, Bonferroni-adjusted *P*=.05/6=.0083). The following single-item fit analysis showed that the personal factor knowledge about spinal cord injury did not fit well (χ^2^_2_=19.1, *P*<.001). After deleting that item, the component displayed satisfactory overall model fit, with a nonsignificant χ^2^ test result for the item-trait interaction (χ^2^_10_=13.6, *P*=.19, Bonferroni-adjusted *P*=.05/5=.01). The means and standard deviations of the fit residuals for items and persons were both within the acceptable limits. The single-item fit analyses for the remaining 5 categories were also satisfactory, with nonsignificant χ^2^ test results (Table 4). The person separation index of this component was 0.68, and no differential item functioning was detected for any of the categories by gender, age, education level, or etiology.

**Table 4:** International Classification of Functioning, Disability, and Health categories retained after multiple rounds of Rasch analysis.

ICF^a^ category			Location	Fit residual	χ^2^^b^	*P* value	Transformation
**Body functions and body structures**							
	1	Sleep functions (b134)	–1.450	–0.762	1.0	.61	4
	2	Emotional functions (b152)	–1.281	–1.414	7.9	.02	3
	3	Sensation of pain (b280)	0.308	1.047	2.4	.30	4
	4	Blood vessel functions (b415)	2.034	0.395	3.7	.16	2
	5	Increased blood pressure (b4200)	1.932	0.789	3.6	.16	3
	6	Decreased blood pressure (b4201)	0.757	–1.106	1.6	.46	3
	7	Immunological system functions (b435)	1.625	–0.922	1.8	.40	2
	8	Respiration functions (b440)	1.767	–0.156	0.8	.68	3
	9	Weight maintenance functions (b530)	0.729	0.523	2.4	.30	1
	10	Sexual functions (b640)	–2.588	–2.086	5.5	.07	2
	11	Procreation functions (b660)	–1.640	–0.862	1.6	.44	2
	12	Mobility of joint functions (b710)	–0.895	–0.791	1.7	.43	2
	13	Muscle power functions (b730)	–2.610	–0.216	3.5	.17	1
	14	Muscle tone functions (b735)	–1.026	–0.586	2.7	.25	4
	15	Structure of areas of skin (s810)	2.339	0.046	1.2	.54	4
**Activities and participation^c^**							
	16	Changing basic body position (d410)	0.252	–1.543	5.6	.06	4
	17	Moving around using equipment (d465)	–0.614	–0.274	2.2	.33	4
	18	Washing oneself (d510)	–1.697	–1.594	5.2	.08	4
	19	Caring for body parts (d520)	1.147	–0.637	0.8	.66	4
	20	Regulating urination (d5300)	–0.866	3.353	8.5	.01	4
	21	Regulating defecation (d5301)	–1.841	0.713	0.4	.80	4
	22	Dressing (d540)	0.127	0.26	0.7	.72	4
	23	Eating (d550) and drinking (d560)	3.493	–0.741	1.3	.52	4
**Contextual factors^d^**							
	24	Assistive products and technology for personal indoor and outdoor mobility and transportation (e1201)	1.805	0.585	2.4	.30	2
	25	Design, construction, and building products and technology of buildings for private use (e155)	–0.177	1.793	2.7	.26	2
	26	Acceptance of life in a wheelchair/in bed	–0.237	–1.264	3.3	.19	2
	27	Coping with everyday life	–1.127	1.305	1.9	.39	2
	28	Adjustment to new body image	–0.263	–0.787	3.3	.19	2

^a^ICF: International Classification of Functioning, Disability, and Health.

^b^Goodness of fit of each category was tested using a *χ^2^* test with a Bonferroni-adjusted *P* value. All were <.01.

^c^Family relationships (d760) and socializing (d9205) were deleted because of poor fit.

^d^Knowledge about spinal cord injury was deleted because of poor fit.

## Discussion

### Principal Findings

The results of the Rasch analysis showed good fit to the Rasch model for the different components of the ICF set as implemented in the app after modification. Both overall and single-item fit were satisfactory. There was no differential item functioning for any of the ICF categories by gender, age, education level, or etiology. These results indicate the suitability of the app-based ICF set as an assessment tool for assessing the functioning of SCI patients.

The app-based ICF set is one of many forms of ICF-based electronic health records. Several previous studies have confirmed the role of ICF-based electronic health records in reflecting patient functioning and facilitating rehabilitation [6,34]. As a unified and standard language originally developed for multidisciplinary use, the original ICF was relatively easily understood by different disciplines and suitable for multidisciplinary teamwork [2]. However, some obstacles still existed, including the complexity of the terminology, lack of operationalization of the ICF qualifiers, and training overload for ICF users. The satisfactory internal construct validity of the set developed in the study is mainly due to the selection of suitable ICF categories and standardized assessment enabled by the app. In this study, the app applied many fewer ICF categories (31) than the comprehensive ICF core set (168 categories) and the brief core set (33 categories) for SCI patients issued by the WHO’s ICF research branch [35]. Additionally, the categories were more specific because they were originally identified as good outcome indicators for SCI patients in China [20,26]. Each ICF category focuses on a specific area and is independent from the others, which helped to ensure the overall fit to the Rasch model based on the assessments’ content.

Together’s verbal prompts standardize assessment and give more consistent assessment results. The transformation guidelines operationalize the 5 ICF qualifiers simply and effectively. With the help of the app, the ICF qualifiers can automatically be matched to the initial clinical assessment results. No additional training on ICF terminology or qualifiers is needed. The process reduces the differences among assessors and makes presentation of the ICF data more convenient and intelligent.

Family relationships (d760), socializing (d9205), and knowledge about spinal cord injury were deleted. In a previous study, d760 and d9205 also exhibited poor model fit [13]. Family relationships are the basis for good functioning in the family, and socializing reflects a patient’s social participation. Both of them are influenced by many factors such as age, severity of the injury, and financial considerations [36,37]. For an item to have perfect fit to the Rasch model, no other factors should influence a person’s performance regarding the item except for the person’s ability and the item’s difficulty, which may explain why the two factors did not fit the Rasch model. The ICF does not classify personal factors because of the large social and cultural variance associated with them [1]. The personal factors assessed in this ICF set were identified by multiround expert surveys [26]. Knowledge about SCI is a broad concept covering many aspects such as injury outcomes, functional rehabilitation, and preventing complications, and it is influenced by multiple factors such as the patient’s level of education, efficacy self-perceptions, and any health education they have received [38]. This may be why the item did not fit the Rasch model well. However, family relationships or support, social participation and the knowledge about SCI are important indicators for the outcomes of rehabilitation. To ensure the completeness of a patient’s health records, it is suggested that they be retained as part of the basic health data collection but not as part of the assessment instrument. Further research should use professional measurements that reflect family relationships, socializing, and the knowledge level for SCI patients.

It is worth noting that, in contrast to the person separation index of the activities and participation component, the person separation index of the other two components was not ideal (0.5 and 0.68), which reflects the poor internal consistency of the two components. This may be related to the different measuring guidelines used. Four guidelines were used in the study to transform the input of the medical staff, based on patient information in the form of percentages, wording from patient reports, frequency with which a problem was observed, and scores on routine clinical instruments or standards. A previous study [29] found that the categories involving guideline 4 (which transforms scores of a routine clinical instrument or standards into the ICF qualifiers) had the best interrater reliability, and the categories involving guideline 2 (which transforms the wording from patient reports into the ICF qualifiers) had the lowest interrater reliability. In this study, all the activity and participation categories used the guideline with the highest reliability (guideline 4), while all the contextual factor categories used the guideline with the lowest reliability (guideline 2). Although the app automatically transforms the initial data into the ICF qualifiers, subjectivity and uncertainty still existed in the patients’ initial reports and recall, which may explain the unsatisfactory person separation index of the two components.

### Limitations

In interpreting these results, it is important to keep in mind that the relatively small sample may have influenced the representativeness of the results. Also, although the app was designed to assess SCI patients at home during transitional care via its remote follow-up function, the study data were collected in face-to-face interviews before the participants were discharged. Further validation with larger samples and remote assessment via the app’s communication function are needed.

### Conclusions

This study has confirmed the suitability of the Together app as an assessment tool. With relatively fewer but more specific ICF categories, it overcomes some of the limitations related to applying the ICF and makes assessment results more reliable and consistent. The app opens up a new way to use the ICF with SCI and electronic health records. In the future, the app could be used to capture information about the functioning of SCI patients at home remotely. Such assessment results would help to monitor patients’ functional changes and differences, learn their needs, identify their problems, and provide evidences for further interventions if necessary.

## Appendix

Multimedia Appendix 1 Together tutorial.

## Abbreviations

ICF: International Classification of Functioning, Disability, and Health
NRS: numerical rating scale
SCI: spinal cord injury
WHO: World Health Organization

## References

[1] (2001). International Classification of Functioning, Disability and Health.

[2] Tempest S, Harries P, Kilbride C, De Souza L (2013). Enhanced clarity and holism: the outcome of implementing the ICF with an acute stroke multidisciplinary team in England. Disabil Rehabil.

[3] Nilmart P, Vongsirinavarat M, Somprasong S, Apinonkul B (2019). Development of an extensive assessment list for knee osteoarthritis based on the International Classification of Functioning, Disability and Health: a Delphi study. Int J Rehabil Res.

[4] Kisser U, Adderson-Kisser C, Coenen M, Stier-Jarmer M, Becker S, Sabariego C, Harréus U (2017). The development of an ICF-based clinical guideline and screening tool for the standardized assessment and evaluation of functioning after head and neck cancer treatment. Eur Arch Otorhinolaryngol.

[5] Vreeman DJ, Richoz C (2015). Possibilities and implications of using the ICF and other vocabulary standards in electronic health records. Physiother Res Int.

[6] Stucki G, Bickenbach J (2017). Functioning: the third health indicator in the health system and the key indicator for rehabilitation. Eur J Phys Rehabil Med.

[7] Bautz-Holter E, Sveen U, Cieza A, Geyh S, Røe C (2008). Does the International Classification of Functioning, Disability and Health (ICF) core set for low back pain cover the patients' problems? A cross-sectional content-validity study with a Norwegian population. Eur J Phys Rehabil Med.

[8] Hilfiker R, Obrist S, Christen G, Lorenz T, Cieza A (2009). The use of the comprehensive International Classification of Functioning, Disability and Health Core Set for low back pain in clinical practice: a reliability study. Physiother Res Int.

[9] Starrost K, Geyh S, Trautwein A, Grunow J, Ceballos-Baumann A, Prosiegel M, Stucki G, Cieza A (2008). Interrater reliability of the extended ICF core set for stroke applied by physical therapists. Phys Ther.

[10] Uhlig T, Lillemo S, Moe RH, Stamm T, Cieza A, Boonen A, Mowinckel P, Kvien TK, Stucki G (2007). Reliability of the ICF Core Set for rheumatoid arthritis. Ann Rheum Dis.

[11] Cao R, Xu G, Ding X, Lin F, Li J (2011). Study on the reliability and validity of international classification of function, disability and health brief core sets for stroke. Chin J Rehab Med.

[12] Gao Y, Yan T, You L, Li K (2018). Developing operational items for the International Classification of Functioning, Disability and Health Rehabilitation Set: the experience from China. Int J Rehabil Res.

[13] Li K, Yan T, You L, Xie S, Li Y, Tang J, Wang Y, Gao Y (2018). Psychometric properties of the International Classification of Functioning, Disability and Health set for spinal cord injury nursing based on Rasch analysis. Disabil Rehabil.

[14] Barton AJ (2012). The regulation of mobile health applications. BMC Med.

[15] Kaplan AL, Cohen ER, Zimlichman E (2017). Improving patient engagement in self-measured blood pressure monitoring using a mobile health technology. Health Inf Sci Syst.

[16] Semple JL, Sharpe S, Murnaghan ML, Theodoropoulos J, Metcalfe KA (2015). Using a mobile app for monitoring post-operative quality of recovery of patients at home: a feasibility study. JMIR Mhealth Uhealth.

[17] Wang Q, Zhao J, Huo X, Wu L, Yang L, Li J, Wang J (2018). Effects of a home care mobile app on the outcomes of discharged patients with a stoma: a randomised controlled trial. J Clin Nurs.

[18] Dahlberg K, Jaensson M, Nilsson U (2019). “Let the patient decide”: person-centered postoperative follow-up contacts, initiated via a phone app after day surgerycondary analysis of a randomized controlled trial. Int J Surg.

[19] De La Cruz Monroy MFI, Mosahebi A (2019). The use of smartphone applications (apps) for enhancing communication with surgical patients: a systematic review of the literature. Surg Innov.

[20] Liu T, Li K, Xie S, Wang Y, Tang J, He X, Yan T (2019). Development of the APP for transitional care of people with spinal cord injury based on the ICF. Chinese J Rehab Med.

[21] Zhang N, Zhou M, Liu N, Liu X, Zhang Y (2018). An investigation study on quality management of spinal cord injury rehabilitation in China 2016. Chinese J Rehab Med.

[22] Zeng X, Wang X, Xu J, Chen T, Xu J (2010). Investigation on the rehabilitation of patients with amputees, spinal cord injuries and craniocerebral injuries after the earthquake in Shifang, Sichuan province. Chinese J Rehab Med.

[23] Chen B, Jiang C, Niu W (2017). Research status of rehabilitation needs of patients with spinal cord injury in Wenchuan earthquake. Chinese J Hygiene Rescue.

[24] Xie H, Yang Y, Wu A, Shen C, Hu L, Zhang J, Lin P, Chen G, Lv J, Chang F (2019). Status quo and development of hope houses for individuals with spinal cord injury in Shanghai from perspective of managers and organizers. Chinese J Rehab Theory Pract.

[25] SunYat-Sen University (2018). Distance transitional care system for spinal cord injury patients. Copyright Protection Center of China Nov2018SR904705.

[26] Li K, Xie S, Wang Y, Tang J, He X, Liu T, Yan T (2019). Outcome indicators in the transitional care of people with spinal cord injury in China: a Delphi survey based on the International Classification of Functioning, Disability and Health. Disabil Rehabil.

[27] Tesio L (2003). Measuring behaviours and perceptions: Rasch analysis as a tool for rehabilitation research. J Rehabil Med.

[28] Schuld C, Franz S, Brüggemann K, Heutehaus L, Weidner N, Kirshblum SC, Rupp R, EMSCI study group (2016). International standards for neurological classification of spinal cord injury: impact of the revised worksheet (revision 02/13) on classification performance. J Spinal Cord Med.

[29] Li K, Yan T, You L, Xie S, Li Y, Tang J, Wang Y, Gao Y (2016). The inter-rater reliability of the International Classification of Functioning, Disability and Health set for spinal cord injury nursing. Int J Rehabil Res.

[30] Grill E, Stucki G (2009). Scales could be developed based on simple clinical ratings of International Classification of Functioning, Disability and Health Core Set categories. J Clin Epidemiol.

[31] Bland JM, Altman DG (1995). Multiple significance tests: the Bonferroni method. BMJ.

[32] Cieza A, Hilfiker R, Boonen A, Chatterji S, Kostanjsek N, Ustün BT, Stucki G (2009). Items from patient-oriented instruments can be integrated into interval scales to operationalize categories of the International Classification of Functioning, Disability and Health. J Clin Epidemiol.

[33] Tennant A, Conaghan PG (2007). The Rasch measurement model in rheumatology: what is it and why use it? When should it be applied, and what should one look for in a Rasch paper?. Arthritis Rheum.

[34] Maritz R, Aronsky D, Prodinger B (2017). The International Classification of Functioning, Disability and Health (ICF) in electronic health records. a systematic literature review. Appl Clin Inform.

[35] (2012). ICF-based documentation tool: ICF Core Sets in clinical practice.

[36] Noreau L, Fougeyrollas P (2000). Long-term consequences of spinal cord injury on social participation: the occurrence of handicap situations. Disabil Rehabil.

[37] Kennedy P, Lude P, Taylor N (2006). Quality of life, social participation, appraisals and coping post spinal cord injury: a review of four community samples. Spinal Cord.

[38] Zhang W, Li S, Li X, Chen C, Tang L, Hu J, Chen D, Wang F, Wang X, Luan R (2019). [Currency survey and influencing factors analysis on the knowledge and behavior related to brucellosis among occupational workers in Jianyang City]. Sichuan Da Xue Xue Bao Yi Xue Ban.

